# Spontaneous Rupture of the Urinary Bladder: A Case Report and Review of the Literature

**DOI:** 10.7759/cureus.112810

**Published:** 2026-07-16

**Authors:** Ekaterina Lazhovska, Gordan Lenart, David Lukanović, Ana Pflaum, Nataša Kenda Šuster

**Affiliations:** 1 Department of Gynecology, Division of Gynecology and Obstetrics, Ljubljana University Medical Centre, Ljubljana, SVN; 2 Department of Urology, Ljubljana University Medical Centre, Ljubljana, SVN; 3 Faculty of Medicine, University of Ljubljana, Ljubljana, SVN

**Keywords:** acute abdomen, acute kidney injury, bladder rupture, hematuria, illicit psychoactive substances, peritonitis

## Abstract

Rupture of the urinary bladder in the absence of trauma is an uncommon condition with a challenging diagnosis that carries a high mortality rate and usually demands immediate surgical intervention. It is commonly associated with underlying chronic bladder pathologies, such as obstructive uropathy, chronic inflammation, and radiation cystitis; in younger patients, it can occur in the setting of substance abuse. Due to its rarity and nonspecific clinical presentation, which often mimics other more common conditions such as bowel perforation, diagnosis is often delayed and made intraoperatively. We report a rare case of atraumatic bladder rupture in a 45-year-old woman presenting with a 14-day history of abdominal pain. Diagnostic laparoscopy performed for suspected peritonitis revealed a 5-7 cm horizontal rupture of the urinary bladder, which was repaired by primary suturing. The exact cause of the rupture could not be definitively established; however, substance abuse was considered a possible contributing factor, as it was the only identifiable risk factor. This case highlights the importance of considering this condition in high-risk patients presenting with an unexplained acute abdomen, particularly when associated with hematuria and renal impairment.

## Introduction

Rupture of the urinary bladder is a rare but potentially fatal condition resulting from disruption of the bladder wall, with subsequent leakage of urine into the surrounding tissues [1]. As the urinary bladder is anatomically well protected by the pelvis, such injuries are most commonly associated with pelvic fractures resulting from high-energy abdominal or pelvic trauma [2]. Penetrating injuries are considerably less frequent [3]. Bladder injuries may also be iatrogenic, caused by mechanical or thermal damage during colorectal, gynecological, or urological procedures, as well as during minor interventions such as urinary catheter insertion [2].

When rupture of the bladder wall occurs in the absence of external trauma, it is referred to as spontaneous rupture of the urinary bladder (SRUB) [1]. SRUB is exceedingly rare, with an estimated incidence of approximately one in 126,000, accounting for less than 1% of all bladder injuries [4]. It usually occurs at the bladder dome, the thinnest and least supported part of the bladder wall, resulting in intraperitoneal rupture [2]. Reported risk factors include previous bladder manipulation, lower urinary tract obstruction [5], chronic bladder inflammation [4], and prior pelvic radiotherapy [5]. In younger patients, spontaneous rupture has been reported in association with substance abuse [5]. Substance abuse may cause urinary retention and bladder overdistension through detrusor relaxation, increased sphincter tone, and suppression of the micturition reflex, potentially leading to bladder rupture. The clinical presentation of SRUB is usually nonspecific, mimicking more common causes of acute abdomen, leading to delayed or missed diagnosis [1]. Overall, it has a poor prognosis with a high mortality rate [6].

We present a rare case of unexplained SRUB in a middle-aged woman, in whom substance abuse was the only identifiable risk factor and may have contributed to the bladder rupture. Drug-associated SRUB remains poorly documented and poses a significant diagnostic challenge because of its rarity. This case highlights the heterogeneous pathophysiology of atraumatic bladder rupture and the importance of maintaining a high index of suspicion in at-risk patients presenting with acute abdominal symptoms.

## Case presentation

A 45-year-old Caucasian woman with a medical history of arterial hypertension treated with a calcium channel blocker, chronic obstructive pulmonary disease, and previous conservatively managed pneumothoraces presented to the ED in the evening with a 14-day history of persistent suprapubic pain that had acutely worsened overnight. She had experienced multiple episodes of nonbloody, nonbilious vomiting during the preceding two weeks, without associated fever or chills, and denied any changes in bowel or urinary habits. She reported the onset of menstruation on the day of presentation and an episode of abnormal uterine bleeding 14 days earlier. She disclosed a single episode of unprotected sexual intercourse one month before presentation.

On examination, the patient was hemodynamically stable, with no signs of dehydration, and in moderate distress secondary to abdominal pain. Physical examination revealed a diffusely tender abdomen without peritoneal signs. Her WBC count and CRP level were only minimally elevated, and the serum pregnancy test was negative. However, laboratory studies showed an elevated creatinine level (203 µmol/L). Relevant laboratory values are shown in Table 1. Conventional abdominal ultrasonography demonstrated a significant amount of free intraperitoneal fluid and an irregular extracavitary soft-tissue mass superior to the uterine fundus, measuring 60 × 45 × 40 mm. The lesion was heterogeneous and partially cystic, possibly arising from the left adnexa. There were no signs of hydronephrosis. Despite the administration of multiple analgesic agents, including opioids, the patient’s pain persisted. However, her vital signs remained within the normal range, with no signs of peritoneal irritation. Analgesic therapy was continued.

**Table 1:** Relevant laboratory findings during hospitalization. Admission laboratory values were obtained at presentation to the ED. Preoperative laboratory values were obtained the following morning. Postoperative laboratory values were obtained on postoperative day 1. CKD-EPI, Chronic Kidney Disease Epidemiology Collaboration; eGFR, estimated glomerular filtration rate

Laboratory parameter	Unit	Day 0 - ED admission	Day 1 - Preoperative	Day 2 - Postoperative	Reference range
Leukocyte count	× 10⁹/L	11	6.4	4.2	4.0-10.0
Hemoglobin	g/L	133	121	95	120-150
Platelet count	× 10⁹/L	304	238	185	150-410
CRP	mg/L	12	94	213	<5
Potassium	mmol/L	4.9	4.2	4.2	3.8-5.5
Sodium	mmol/L	132	130	136	135-145
Creatinine	µmol/L	203	216	44	49-90
eGFR (CKD-EPI)/1.73 m²	mL/min	25	23	>90	≥90

The patient was then referred from the abdominal to the gynecological ED with suspected pelvic inflammatory disease (PID) complicated by a tubo-ovarian abscess (TOA). Further targeted history revealed that the patient had, in fact, been passing less urine than usual. Additional evaluation demonstrated marked adnexal tenderness on palpation, further supporting the differential diagnosis of PID. The patient was admitted that evening to the Department of Gynecology for further evaluation and management.

Intravenous analgesic and antibiotic therapy were initiated immediately. The acute kidney injury (AKI) was considered prerenal, given the history of profuse vomiting. Additional laboratory evaluation did not reveal significant electrolyte disturbances or metabolic acidosis, except for mild hyponatremia; therefore, isotonic saline was selected for fluid therapy. After insertion of a Foley catheter for fluid balance monitoring, mild gross hematuria was noted. Following initiation of treatment, the patient’s pain improved and remained adequately controlled with minimal analgesic requirements. She remained hemodynamically stable throughout the night.

**Figure 1:**
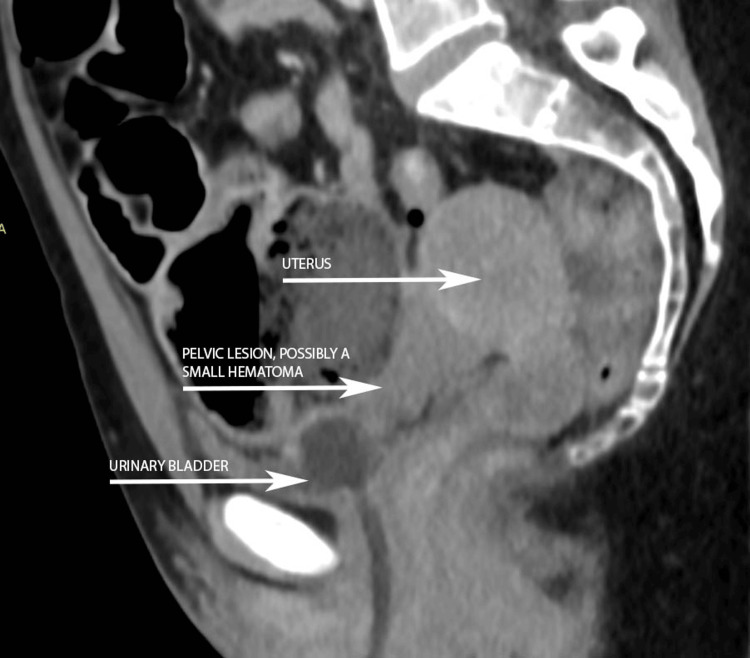
CT of the abdomen revealing a 25 × 60 mm lesion above the urinary bladder, consistent with a hematoma.

A follow-up pelvic ultrasound the next morning showed no pathological findings in the gynecological organs and, surprisingly, no free intraperitoneal fluid. However, a mass consistent with a blood clot, measuring approximately 50 mm, was identified in the pelvis. Repeat laboratory investigations revealed worsening renal function and increased CRP levels (Table 1). Because the pain worsened the following morning and the ultrasound findings were inconsistent with the working diagnosis, an urgent contrast-enhanced abdominal CT scan was obtained. It revealed free intraperitoneal air suggestive of hollow viscus perforation; however, the site of perforation could not be identified. A pelvic lesion measuring approximately 25 × 60 mm, consistent with a small hematoma or an endometriotic focus, was also noted (Figure 1). There were no intra-abdominal or pelvic abscesses. 

A diagnostic laparoscopy was indicated based on the CT findings. During the preoperative assessment and examination, signs of peritonitis were present. During this assessment, a nasal deformity was noted; together with episodes of unusual behavior, this raised suspicion of possible substance abuse. A review of the patient’s electronic medical records then revealed a documented history of substance use disorder, with multiple prior hospital admissions for intoxication involving alcohol, cocaine, and gamma-hydroxybutyrate.

**Figure 2:**
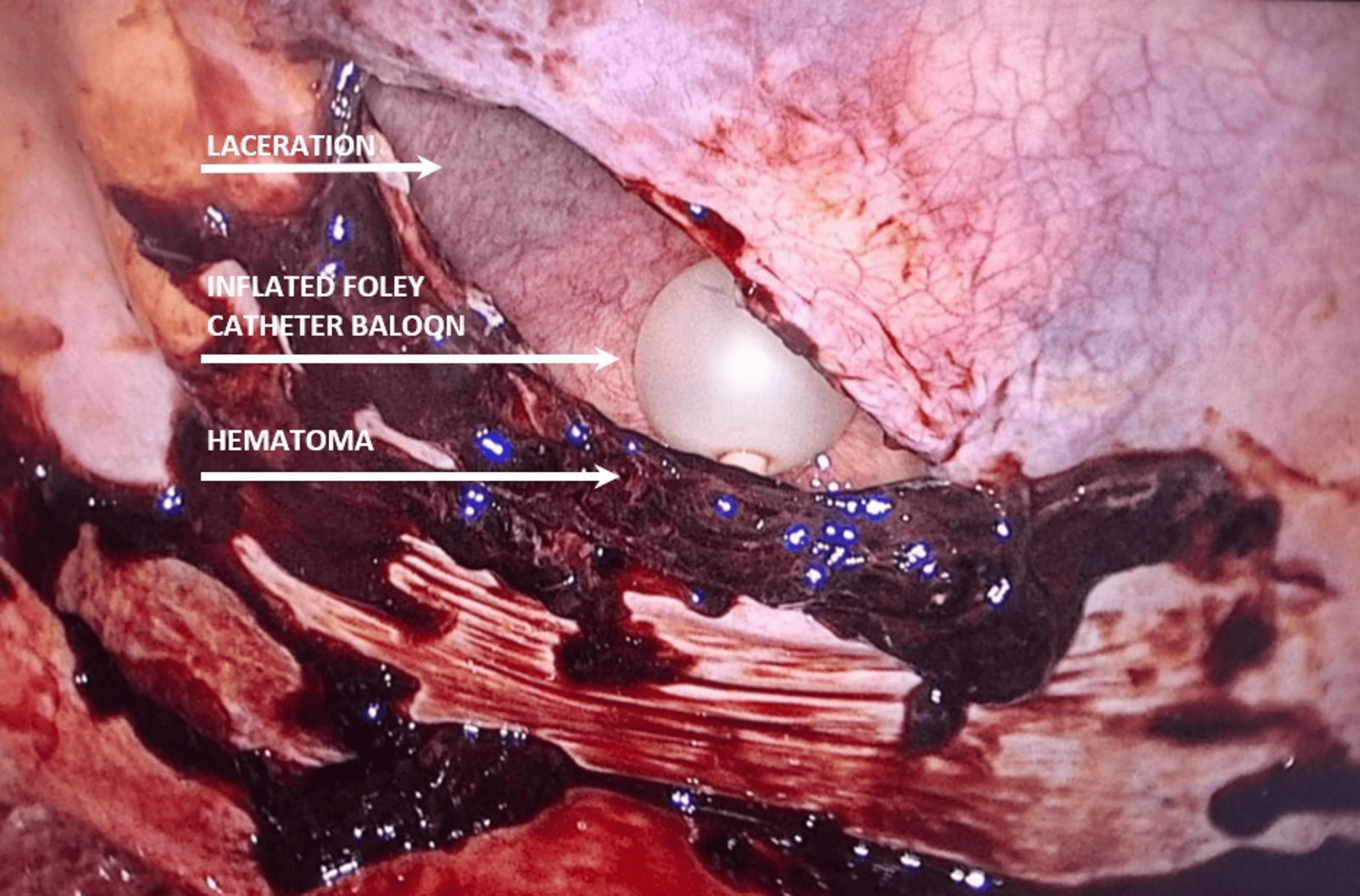
Intraoperative view demonstrating a 5-7 cm laceration of the bladder wall, partially covered by a blood clot, with the Foley catheter balloon visible within the bladder lumen.

An emergency diagnostic laparoscopy was performed that evening, revealing a 5-7 cm-long horizontal transmural laceration at the apex of the bladder dome, covered by a blood clot (Figure 2).

A urology consultation was obtained intraoperatively. The procedure was converted to an open laparotomy, the bladder wall was closed by primary suturing, and an abdominal drain was placed. Empiric antimicrobial therapy with amoxicillin-clavulanic acid was initiated, and the previous antimicrobial therapy for suspected PID was discontinued. On the following day, CRP levels increased, whereas the serum creatinine level had returned to normal (Table 1). The remainder of the postoperative course was uneventful. Microbiological cultures obtained preoperatively and intraoperatively yielded no growth. The patient was discharged home in stable condition on postoperative day 7 with a Foley catheter in place. Normal bladder integrity was confirmed by cystography on postoperative day 12, after which the catheter was removed.

During outpatient follow-up, the patient reported only occasional mild discomfort in the lower abdomen, and laboratory and ultrasonographic findings were unremarkable.

## Discussion

Etiology

Rupture of the bladder wall in the absence of external trauma is an extremely rare clinical entity [4]. SRUB can occur because of several predisposing factors; it is typically caused by an acute rise in intravesical pressure in the setting of a weakened bladder wall [7], which is the main risk factor for SRUB [7]. One of the most common underlying causes is chronic bladder outlet obstruction [8], due to urethral strictures, tumors, pelvic organ prolapse in women, or benign prostatic hyperplasia in men [7]. Other etiological factors contributing to bladder wall weakness include mechanical damage, such as prolonged catheterization [9], local tumor infiltration [7], and chronic inflammation resulting from recurrent cystitis of bacterial, fungal, or tuberculous origin [10]. Chronic bladder irritation leads to detrusor hypertrophy and fibrotic remodeling of the bladder wall. Over time, the bladder wall becomes less elastic and thinner, making it susceptible to rupture during an acute rise in intravesical pressure [7], for example, during acute urinary retention or acute bladder outlet obstruction. Spontaneous bladder rupture is considered less common in women, likely because of the lower resistance of the bladder neck and urethra. Women usually leak urine before the bladder ruptures [8].

Radiation cystitis is a well-recognized risk factor for bladder rupture that develops in approximately 5-10% of patients following pelvic radiotherapy for malignancies of the uterus, rectum, prostate, or bladder [11]. Radiation can cause damage ranging from mild inflammation to ulceration and necrosis [9], as well as reduced compliance of the bladder wall [11], all of which may lead to bladder rupture [9]. Associated neuropathy due to surgery-related nerve injury can also be a contributing factor. Radiation-induced bladder rupture usually occurs more than a decade after completion of radiotherapy; according to the literature, the incidence following radiotherapy for gynecological cancers is approximately 2.1%. Morita et al. described a case of atraumatic bladder rupture in a 78-year-old woman that occurred 37 years after adjuvant radiotherapy for cervical cancer. The patient had been performing intermittent self-catheterization for urinary retention. Emergency laparoscopy, performed for suspected ileus, revealed strangulation ileus with a bowel loop incarcerated within the perforated bladder wall [11].

Kabarriti et al. reported a rare case of spontaneous bladder rupture as a complication of diabetic cystopathy in a 36-year-old patient [6]. Diabetic cystopathy is an uncommon manifestation of diabetes mellitus [7] that may occur at any stage of the disease, including the early stages [6]. It is characterized by increased postvoid residual volumes, reduced bladder sensation, and impaired detrusor contractility due to diabetes-associated neuropathy [12]. Poor glycemic control increases the incidence of urinary tract infections, which is an additional risk factor for bladder wall vulnerability [4].

While spontaneous bladder rupture usually occurs in older patients with chronic bladder pathology, it can also occur in younger individuals without known underlying bladder pathology, most commonly in the context of acute alcohol intoxication. The combination of increased urine production, diminished perception of bladder fullness, and altered consciousness can lead to rapid bladder overdistension and progressive thinning of the bladder wall, predisposing it to rupture [5]. Even minimal increases in intra-abdominal pressure, such as during coughing, may cause bladder perforation [10]. A comprehensive review of 84 published articles identified alcohol abuse as the causative factor in 25 cases of atraumatic bladder rupture [13].

Alcohol abuse frequently coexists with the use of illicit psychoactive substances, which may further contribute to bladder rupture [8]. Alpha-sympathomimetic agents, including cocaine and methamphetamine, exert adrenergic effects on the richly innervated urethral sphincter. This leads to increased sphincter tone and impaired bladder emptying [8], thereby raising intravesical pressure and the risk of rupture [1]. It is unlikely that cocaine-associated SRUB is mediated by a direct local anesthetic effect on the bladder mucosa, because only 1-9% of cocaine is excreted unchanged in the urine [8].

Opioids, on the other hand, induce detrusor relaxation and suppress visceral afferent signaling. Animal studies have demonstrated marked inhibition of the micturition reflex during bladder distension, resulting in impaired perception of bladder fullness and an increased susceptibility to spontaneous rupture. Galbraith et al. reported a case of SRUB in a 24-year-old woman who presented with lower abdominal pain that began abruptly during micturition. Over the preceding few days, she had ingested codeine, probably leading to urinary retention. Diagnostic laparoscopy, performed because of deterioration of her condition, revealed a perforation of the bladder wall. The injury was believed to be caused by increased intra-abdominal pressure acting on the distended bladder during the attempt to void [1].

Other illicit substances, such as cannabis and ketamine, have been associated with urinary retention and ulcerative cystitis, which, in theory, could predispose patients to atraumatic bladder rupture [14,15]. However, no cases of bladder rupture attributable to substances other than opioids and alpha-sympathomimetic agents have been reported in the available literature.

In our case, the exact cause of SRUB could not be definitively established. The patient had a well-documented history of cocaine and alcohol use and no other recognized risk factors for spontaneous bladder rupture. Substance use may have contributed to bladder rupture through the mechanisms described above. As patients often conceal substance use, establishing the diagnosis may be particularly challenging, further delaying recognition. In retrospect, toxicology screening could have provided valuable additional information and represents a limitation of this case.

Clinical presentation and diagnosis

Bladder rupture resulting from increased intravesical pressure usually occurs at the bladder dome, which is the thinnest region of the bladder, making SRUB an intraperitoneal injury. In contrast, traumatic bladder injuries are typically extraperitoneal and involve the anterior or lateral bladder wall [2]. Some rare cases of extraperitoneal SRUB have also been described [8].

In intraperitoneal rupture, urine enters the peritoneal cavity, leading to chemical peritonitis and causing nonspecific symptoms such as diffuse abdominal pain, nausea, and vomiting. Pain is usually more severe in the suprapubic region [8]. Accumulation of urine can cause abdominal distension [7], as well as shoulder pain due to diaphragmatic irritation [8]. Although a classic triad consisting of abdominal pain, oliguria or anuria, and gross hematuria is considered highly suggestive of intraperitoneal bladder rupture [7], it is rarely present in clinical practice. In our case, all three features were present; however, the diagnosis was not initially recognized. The patient’s reduced urine output was initially attributed to dehydration secondary to prolonged vomiting, whereas gross hematuria was presumed to be related to menstrual bleeding. Bladder rupture is often misdiagnosed as AKI [5], as peritoneal reabsorption of creatinine and urea can produce a biochemical picture mimicking acute renal failure [1,5,10]. However, it remains unclear whether the observed AKI in our case was entirely pseudo-renal or represented true prerenal kidney injury related to prolonged vomiting over the preceding two weeks. Bladder rupture should be considered in patients presenting with an acute abdomen and unexplained renal impairment, especially when hematuria is also present.

Additionally, in our patient, Foley catheter insertion was followed by a marked increase in urine output, which was initially attributed to increased fluid administration in the setting of AKI. However, the follow-up ultrasound performed the following morning, within 12 hours of catheter insertion, showed no intra-abdominal fluid. This finding should have raised suspicion for bladder wall disruption, as the resolution of the excessive free intraperitoneal fluid was probably due to bladder drainage. Although the patient reported abdominal pain and vomiting for two weeks before admission, we consider it unlikely that bladder rupture had been present throughout this period because of the initially only minimally elevated inflammatory markers and her hemodynamic stability. We believe that the patient may have experienced urinary retention and progressive bladder overdistension during the preceding two weeks, with bladder rupture occurring acutely shortly before presentation. Although the patient initially denied any changes in urination, a more detailed history later revealed reduced urine output shortly before admission. Our hypothesis is further supported by the subsequent rise in CRP the day after admission and by the complete normalization of renal function following surgical repair.

Due to its rarity and nonspecific clinical presentation, SRUB is generally not diagnosed preoperatively. One study reported a mean delay in diagnosis of 5.4 days, with a range of two hours to 36 days [1]; in our case, the diagnosis was made within 24 hours of the initial medical assessment. In more than 50% of reported cases [5], the diagnosis is made intraoperatively during exploratory surgery for acute peritonitis [7], which was also the case in our patient.

The initial differential diagnosis commonly includes gastrointestinal pathologies, particularly bowel perforation, as abdominal CT often demonstrates findings suggestive of gastrointestinal perforation, such as pneumoperitoneum. Although free intraperitoneal air is uncommon in isolated intraperitoneal bladder rupture, it may occur following urinary catheterization in the presence of a bladder defect. This was probably the explanation in our case as well.

In our case, the preliminary diagnosis was PID complicated by a TOA, based on the history of recent unprotected sexual intercourse, suspected abnormal uterine bleeding, adnexal tenderness on palpation, and a mass consistent with a TOA on ultrasonography. When bladder rupture is suspected clinically, CT cystography is the diagnostic modality of choice [3].

Treatment

There are no specific treatment guidelines for SRUB; therefore, management should be based on guidelines for traumatic bladder injury. The European Association of Urology (EAU) recommends surgical management of intraperitoneal bladder injuries, with exploratory laparotomy considered the standard approach. The guidelines of the World Society of Emergency Surgery and the American Association for the Surgery of Trauma are the only ones that permit a laparoscopic approach in hemodynamically stable patients. The bladder wall may be repaired using either single- or double-layer closure, followed by continuous bladder drainage with a urinary catheter. The EAU guidelines recommend cystography in complex cases or in patients with risk factors for impaired healing. In contrast, extraperitoneal injuries are generally managed conservatively with Foley catheter drainage; however, complicated injuries should be managed surgically. Conservative management of intraperitoneal injuries may be considered only for small, uncomplicated injuries, which usually occur during endoscopic procedures, and relies on urinary drainage and antibiotic therapy [3].

## Conclusions

SRUB is a rare but serious clinical entity associated with significant morbidity and mortality. Early recognition is essential for successful treatment, which is most often surgical. A high index of suspicion is warranted in patients presenting with an unexplained acute abdomen, particularly those with known bladder dysfunction, risk factors for increased intravesical pressure, or urinary symptoms such as gross hematuria and oliguria. Illicit substance use is increasingly recognized as a risk factor for SRUB, particularly in younger patients.
